# Two Cases of Pyæmia, or Purulent Infection, with Recovery; in Which the Bisulphite of Soda was Administered

**Published:** 1865-02

**Authors:** Walter F. Atlee


### TWO CASES OF PYÆMIA, OR PURULENT INFECTION, WITH RECOVERY; IN WHICH THE BISULPHITE OF SODA WAS ADMINISTERED.

Communicated by WALTER F. ATLEE, M.D.

The history of the following two cases is communicated because I am fully persuaded that they were cases of pyæmia. They were treated by a new method, from which extraordinary results are said to have been obtained in other countries; and by all other treatment, so far as I have seen, well-marked, undoubted cases of pyæmia have invariably proved fatal.

Nélaton says that *pyæmia is always fatal*, and adds that in the cases of cure which some surgeons suppose that they have seen, they allowed themselves to be imposed upon by certain circumstances which may lead, in these cases, to an error in diagnosis. (*Elemens de Path. Chirurg.*, vol. i. p. 167.) The authors of the *Compendium de Chirurgie Pratique* (A. Bérard and Denonvilliers) say that the gravity of a disease, the principal condition of which is the infection of a liquid, destined to circulate through and nourish every part of the body, is at once understood—"*such, in fact, is its gravity, that art has so far remained powerless against it.*"—(p. 386.)

I have thought it proper to cite these authorities as to the invariable fatality of pyæmia, inasmuch as it is supposed by persons of comparatively small experience and powers of observation to be not unfrequently cured. I have seen, myself, a large number of cases of pyæmia, and the two which are here reported are the only ones that recovered.

The treatment instituted in these cases was in accordance with that first recommended and practised by Prof. Polli, and brought to the notice of the profession in this country in the numbers of this Journal for October, 1862 (p. 513 *et. seq.*), and for April, 1863 (p. 467 *et. seq.*). Prof. Polli has succeeded in establishing that not only does sulphurous acid possess the property of arresting fermentation, and neutralizing catalytic action, but that its alkaline and earthy compounds have also the same power, and, moreover, that they can be administered with the greatest impunity, even in large doses. Pyæmia, or purulent infection, which latter name is preferable, though the other must be employed, since it is generally adopted in this country, is a disease that depends essentially on the presence in the blood of an organic poison, which acts as a ferment, and multiplies itself. Until the announcement of the discovery of Prof. Polli, no substance was known that could destroy a catalytic poison, without, at the same time, so altering the blood itself, as to render it incapable of performing its vital functions. Bernard has declared that the neutralization of such poisons is impossible. (*Lecons sur les effets des substances toxiques et medicamenteuses*, p. 99.)

Case I. Reported by Joseph B. Roe, M.D., A.A. Surgeon, U.S.A. Ezra Reagles, private, Co. A, 36th Wisconsin, aged 30, by occupation a farmer, was admitted to Ward 3, Satterlee U.S.A. General Hospital, West Philadelphia, Pa., August 20, 1864, from Field Hospital, City Point, Va. Wounded at Deep Bottom, Va., August 16, 1864, by a minie ball, which fractured metatarsal bones of left foot. Patient and wound in good condition at time of admission, and continued so until August 28th, when gangrene set in. From this date until September 4th, the gangrene rapidly spread, so that nearly the whole of the upper part of the foot was involved. Sugar, which had heretofore been so successfully used in the hospital, in the treatment of all gangrenous wounds, was applied at the first appearance of the gangrene, but to no good purpose. The character of the wound and the condition of the patient became such, that it was decided to amputate the foot, which was accordingly done, September 5th, about three inches above the ankle-joint. The patient did remarkably well until September 12th, when he had a violent chill, and complained of severe pain, upon pressure, over the region of the liver. The chills returned on the 13th, 14th, and 15th. The flaps had, by this time, sloughed away so as to leave about two inches of the bones exposed. At the first appearance of the chills one (1) drop of nitro-muriatic acid, in the infusion of quassia, was administered thrice daily, with beef tea, milk punch, &c., until Sept. 14th, when, at the suggestion of Dr. Walter F. Atlee, eight (8) grains of the bisulphite of soda was ordered every four hours. From the 18th the patient commenced rapidly to improve, and no untoward symptoms made their appearance up to November 8th, 1864, when the patient was transferred to Ward A, in consequence of the vacation of Ward 3. The general health of the patient at this time was remarkably good, the exposed bones had exfoliated, and the stump nearly healed.

Case II. Reported by A. A. Smith, M.D., A.A. Surgeon, U.S.A. Private John C. Friesman, Co. H, 8th New York Heavy Artillery, aged 51, and by occupation a painter, was admitted from Field Hospital, City Point, Va., August 20th, 1864, with a gun-shot flesh wound of both legs, received at the battle near Malvern Hill, Va., August 16th, 1864. The ball—minie—passing transversely through the middle of the middle-third of the left leg, between the soleous and gastrocnemius muscles, producing a severe flesh wound; thence through the middle-third of the right leg, anterior to the tibia, producing a slight flesh wound, and denuding the tibia of periosteum, along the track of the ball. On admission the patient's general health was considerably impaired by the exposure and hardships of active field duty. Wound suppurating very little, and the discharge was of a very unhealthy character. Ordered tonics, stimulants, and water-dressings. Up to October 2d, nothing of importance occurred. The patient's general health seemed to suffer from the suppuration, which became excessive after the twelfth day. On the morning of the 2d, he had a severe chill, followed by slight febrile reaction, then a profuse clammy perspiration, and low, muttering delirium; features blanched and sunken. Oct. 3d, 4th, 5th, 6th. Heavy chills and delirium. The patient apparently sinking rapidly. Oct. 7th, 8th. Chills not so marked; less delirium, and general appearance improved. Oct. 9th. A very light chill, and very little perspiration. Immediately after the appearance of the first chill, on the 2d of October, the bisulphite of soda was given in gr. xx doses every two hours, which was continued until the 12th, after which twenty grains were given twice a-day until the 1st November, when it was stopped. Milk punch, beef essence, and f℥j of the following mixture every two hours were also given: Quinia sulph. gr. xxiv, tinct. ferri chlor. f℥ss, syr. simpl. f℥iss, aqua cinnam. f℥iv. Oct. 12th. A slight rigor; suppuration from wound a little more healthy; general appearance decidedly improved, and some desire for food. Oct. 20th. *Decided* improvement; very little suppuration, and the wound granulating; no return of chill, and the mind perfectly clear since the 12th inst. Nov. 1st. Able to sit up; wound healing rapidly. Nov. 17th. General health much better than when admitted; appetite good, and wound closing rapidly; walks about on crutches.—*Am. Jour. Med. Sciences.*

